# Schuylkill Valley

**Published:** 1896-04

**Authors:** Otto Noack

**Affiliations:** Corresponding Secretary


Secretary.

### SCHUYLKILL VALLEY VETERINARY MEDICAL ASSOCIATION.

The quarterly meeting was held on Wednesday, March 18, 1896, in the room of Common Council, City Hall, Reading. It was called to order at 1 P.M. by President Burkholder. Dr. Noack acted as Recording Secretary. At roll-call the following members responded: Drs. Burkholder, Sallade, McCarthy, Noack, Fegley, Kershner, Brackbill. The minutes of the last meeting were read and approved.

The Committee on Legislation reported as to the communication which should be brought before the councils of the different sections of counties. The members of the Society living in this section were appointed to bring the matter before the different councils and see to carrying it through.

Dr. Sallade, of the Board of Examiners, reported a case where he was asked by an attorney about the registration of a man who was for ten years practising, but did not know the law and consequently did not register. The Association instructed Dr. Sallade to answer in the negative.

On request of Dr. Sallade for support of the Board of Examiners, Dr. McCarthy made the motion that for this purpose an assessment shall be made for the prosecution of illegal practitioners as far the interests of the members of the Association are concerned. The motion was seconded and carried.

Dr. Noack's motion that he should be authorized to write to the Governor for reappointment of Dr. Sallade in the Board of Examiners was carried.

Introduction of initiation fee for new members, and also that words in the by-laws "or existing practitioners" for becoming members should be stricken out, should be brought up before next meeting, because any change of the by-laws has to be made in writing by two members before the next meeting.

The President called upon Dr. Brackbill to read his paper on "Spavin," which was ably discussed by all members present as to the different modes of treatment. In the absence of Dr. Noack, a translation of the *Revue Vétérinaire* about "Inoculation of Tuberculosis of the Gallinæ in the Mammifers" was read by Dr. Fegley. The "General Pathology of the Lymphatic

System" was the subject of the paper read by Dr. McCarthy. Programme for next meeting: Bieber, "Mange;" Moyer, "Castration;" Faughnan, "Tetanus;" Sallade, the "Social Position of the Veterinarian;" Noack, "Emphysema."

Moved to adjourn at 5.30 P.M., and to meet at Pottsville the third Wednesday in June.

OTTO NOACK,
Corresponding Secretary.

